# Analysis of CyberKnife intracranial treatment plans using ICRU 91 dose reporting: A retrospective study

**DOI:** 10.1002/acm2.13932

**Published:** 2023-02-16

**Authors:** Dion Conlon, James Connolly, Mohamed Galal, Ismail Ahmed, Mark Foley, Christoph Kleefeld

**Affiliations:** ^1^ School of Physics University of Galway Galway Ireland; ^2^ Department of Medical Physics Blackrock Health Galway Clinic Galway Ireland; ^3^ Department of Medical Physics Blackrock Health Hermitage Medical Clinic Dublin Ireland

**Keywords:** conformity index, CyberKnife, gradient index, ICRU 91, stereotactic, treatment planning

## Abstract

ICRU 91, published in 2017, is an international standard for prescribing, recording, and reporting stereotactic treatments. Since its release, there has been limited research published on the implementation and impact of ICRU 91 on clinical practice. This work provides an assessment of the recommended ICRU 91 dose reporting metrics for their use in clinical treatment planning. A set of 180 intracranial stereotactic treatment plans for patients treated by the CyberKnife (CK) system were analyzed retrospectively using the ICRU 91 reporting metrics. The 180 plans comprised 60 trigeminal neuralgia (TGN), 60 meningioma (MEN), and 60 acoustic neuroma (AN) cases. The reporting metrics included the planning target volume (PTV) near‐minimum dose ($D_{\mathrm{near}-\min}$), near‐maximum dose ($D_{\mathrm{near}-\max}$), and median dose ($D_{50\%}$), as well as the gradient index (GI) and conformity index (CI). The metrics were assessed for statistical correlation with several treatment plan parameters. In the TGN plan group, owing to the small targets, $D_{\mathrm{near}-\min}$ was greater than $D_{\mathrm{near}-\max}$ in 42 plans, whereas both metrics were not applicable in 17 plans. The $D_{50\%}$ metric was predominantly influenced by the prescription isodose line (PIDL). The GI was significantly dependent on target volume in all analyses performed, where the variables were inversely related. The CI was only dependent on target volume in treatment plans for small targets. The ICRU 91 $D_{\mathrm{near}-\min}$ and $D_{\mathrm{near}-\max}$ metrics breakdown in plans for small target volumes below 1 cm^3^; the Min and Max pixel should be reported in such cases. The $D_{50\%}$ metric is of limited use for treatment planning. Given their volume dependence, the GI and CI metrics could potentially serve as plan evaluation tools in the planning of the sites analyzed in this study, which would ultimately improve treatment plan quality.

## 1 INTRODUCTION

The ICRU (International Commission on Radiation Units and Measurements) has been involved in the continuous effort to improve uniformity in defining terms and concepts and in specifying radiation doses for reporting in radiotherapy.^1^ The commission published ICRU 50 and 60 which address conventional conformal radiotherapy,^2^, ^3^ whereas ICRU 83 covers intensity‐modulated radiotherapy (IMRT).^4^

Stereotactic treatment (ST) combines stereotactic localization techniques with the delivery of multiple small photon beams to conformally irradiate small targets within the body with high doses, while still preserving the surrounding normal tissue^1^, ^5^, ^6^; this condition gives rise to steep dose gradients away from the target. ST is distinct from conventional radiotherapy and IMRT in several aspects, hence there are limitations to the previous ICRU reports (i.e., ICRU 50, 62, and 83) recommendations for prescribing and reporting STs.^7^

An increase in the number of radiotherapy departments embarking on ST programs accentuated the need for an international standard covering these treatments. Hence, in 2017 the ICRU published Report 91 (ICRU 91) on “Prescribing, Recording, and Reporting of Stereotactic Treatments with Small Photon Beams.”^1^

The report provides a framework for reporting radiation doses in STs, as well as a framework for dose prescription. For reporting, it recommends departments provide multiple treatment plan metrics. This includes the following dose metrics: planning target volume (PTV) near‐minimum dose ($D_{\mathrm{near}-\min}$), near‐maximum dose ($D_{\mathrm{near}-\max}$), and median absorbed dose ($D_{50\%}$). In addition, the gradient index (GI) and conformity index (CI) of the plan should also be reported.

Published literature relating to ICRU 91 is scarce, which is probably owed to its relatively recent publication. De Jong et al.^8^ quantified the current clinical practice regarding treatment planning and dose prescription of peripheral lung stereotactic body radiotherapy (SBRT) across eight institutions, by means of the ICRU 91 reporting metrics. Dupic et al.^9^ retrospectively analyzed stereotactic radiotherapy (SRT) plans for brain metastases using the ICRU 91 reporting metrics, and through patient follow‐up, attempted to identify predictive factors of local control.

This study presents an assessment of the clinical use of the ICRU 91 reporting metrics for treatment planning. This assessment was performed through a retrospective analysis of 180 CyberKnife (CK) intracranial treatment plans for three common clinical indications treated at the Hermitage Medical Clinic (HMC), Dublin, Ireland. These indications included trigeminal neuralgia (TGN), meningioma (MEN), and acoustic neuroma (AN).

## 2 METHODS

### 2.1 Patient population and treatment protocols

180 clinical treatment plans for patients previously treated with stereotactic radiosurgery (SRS) or SRT by the CK VSI system (Accuray Inc., Sunnyvale, CA, USA, V9.5) at HMC were incorporated into this study retrospectively. The three treatment sites were chosen to provide a broad range of target sizes and shapes, with TGN cases presenting the smallest possible SRS targets, MEN with larger spherical targets, and AN with irregular shaped targets. The cases were selected from the HMCs patient database and included 60 for each treatment site. The timelines within which these treatments were performed are provided in Table 1.

**TABLE 1:** The treatment timelines and prescription dose regimens from the trigeminal neuralgia, meningioma, and acoustic neuroma groups

Treatment timelines	Trigeminal neuralgia June 2018–January 2021	Meningioma April 2017–April 2021	Acoustic neuroma February 2019–April 2021
Prescription dose regimens	6000cGy/#1 5400cGy/#1	1300cGy/#1 1500cGy/#1 1800cGy/#3 2100cGy/#3 2400cGy/#3 2500cGy/#5	1100cGy/#1 1200cGy/#1 1300cGy/#1 1800cGy/#3 2500cGy/#5

*Note*: The # symbol denotes the number of treatment fractions.

The prescription protocol for TGN treatments at HMC is 6000 cGy in a single fraction,^10^ however, 5400 cGy in a single fraction is also prescribed for TGN retreatments. Thus, from the 60 TGN plans, 56 of them had a prescription dose (PD) of 6000 cGy, whereas the remaining 4 plans had a PD of 5400 cGy. All plans presented 100% coverage of the nerve target (i.e., short segment of the trigeminal nerve with no PTV margin added).

In the MEN group, 15 plans had a gross tumor volume (GTV) contoured only, whereas the remaining 45 plans had a GTV with an additional 1 mm margin added to form a PTV. As per protocol, all plans satisfied the following condition: ≥95% of the GTV/PTV is covered by at least the PD. The PD regimens in the MEN group are presented in Table 1.

All 60 AN plans comprised a GTV with an additional 1 mm margin added to form a PTV, while ≥95% of the PTV was covered by at least the PD. The PD regimens in the AN group are also provided in Table 1.

**FIGURE 1:**
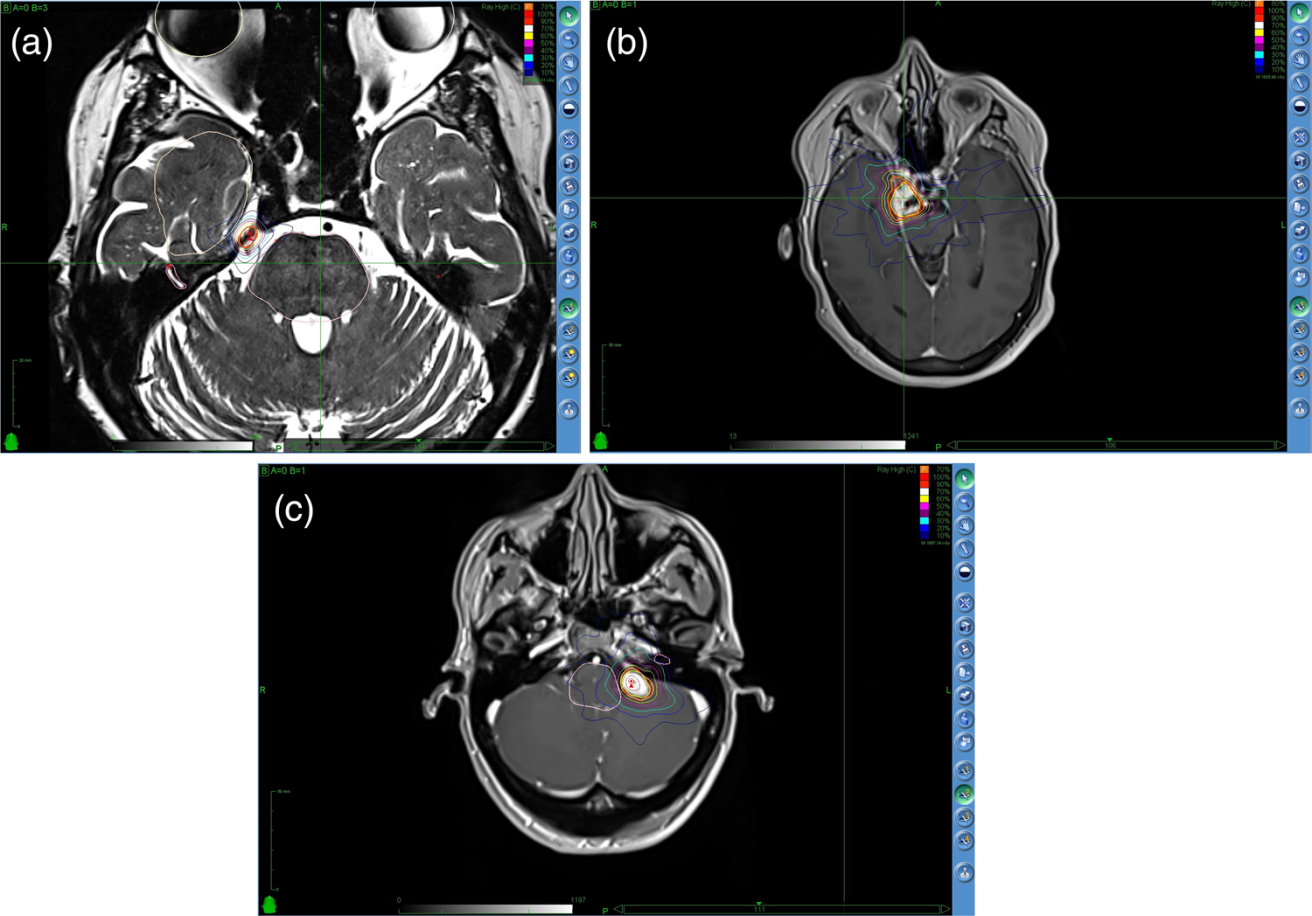
The isodose distribution in the transverse MRI plane from a representative (a) trigeminal neuralgia, (b) meningioma, and (c) acoustic neuroma CyberKnife treatment plan. These treatment plans were prescribed to the (a) 78%, (b) 80%, and (c) 70% isodose line.

Figure 1 shows the isodose distribution of a representative (a) TGN, (b) MEN, and (c) AN plan. A summary of the relevant plan parameters from the three plan groups is provided in Table 2.

**TABLE 2:** Summary of relevant treatment plan parameters from the trigeminal neuralgia, meningioma, and acoustic neuroma groups

Treatment plan parameter	Range	Mean (median)
**Trigeminal neuralgia**		
Target volume (cm^3^)	0.021–0.071	0.043 (0.042)
PIDL (%)	68–85	78 (78)
NOB	106–222	184 (190)
MUs	10 578–20 207	16 618 (17209)
Number of nodes	55–99	86 (89)
HI	1.18–1.47	1.29 (1.28)
Cone collimator sizes (mm)	5–7.5	
**Meningioma**		
Target volume (cm^3^)	0.34–43.48	7.40 (3.78)
PIDL (%)	72–82	79 (79)
NOB	97–306	178 (169)
MUs	7016–54 414	20 374 (14477)
Number of nodes	58–115	86 (86)
HI	1.22–1.39	1.27 (1.27)
Target coverage (%)	95–100	99 (99)
Cone collimator sizes (mm)	7.5–15 (single fraction)	
	7.5–30 (fractionated)	
**Acoustic neuroma**		
Target volume (cm^3^)	0.21–14.03	3.49 (1.88)
PIDL (%)	69–82	76 (78)
NOB	100–293	152 (150)
MUs	3527–43 351	14 593 (12880)
Number of nodes	60–107	82 (83)
HI	1.22–1.45	1.32 (1.28)
Target coverage (%)	95–100	97 (97)
Cone collimator sizes (mm)	7.5–12.5 (single fraction)	
	7.5–20 (fractionated)	

Abbreviations: NOB, number of treatment beams; PIDL, prescription isodose line.

### 2.2 Treatment planning process

For all three treatment sites, the following computed tomography (CT) images (Siemens SOMATOM Definition Edge) were acquired: 120 kVp; 400 mAs; 1 mm slice thickness; without contrast. For TGN cases, these CT images were fused with T1 and T2 magnetic resonance imaging (MRI) images (Siemens MAGNETOM Skyra 3T) (1 mm slice thickness; without contrast), and with additional high resolution Constructive Interference in Steady State (CISS) images (0.5 mm slice thickness; without contrast) for viewing the TGN nerve in detail. For MEN and AN cases, the CT images were fused with volumetric T1 (0.9 mm slice thickness; contrast enhanced) and T2 (1 mm slices thickness; without contrast) images. The GTV/PTV and nearby critical organs at risk (OARs) were delineated by a practicing radiation oncologist and neurosurgeon. The maximum absorbed pixel dose (Max Pixel) to the target was set in line with the required prescription isodose line (PIDL) (i.e., PD as a percentage of the Max pixel) (e.g., 7500 cGy for a TGN case with a PD of 6000 cGy prescribed at 80%); this was set as a hard constraint in the optimization parameters. Avoidance zones where treatment beams are not permitted were set by the planner (e.g., eye structures). The inbuilt automatic cone selection tool in MultiPlan (Accuray Inc., Sunnyvale, CA, USA, V4.6) (i.e., CKs dedicated treatment planning system) was employed by the planner to select the most appropriate cone for the target. The planner adjusts this cone selection, typically selecting slightly smaller cones. A beamset of useful beams from suitable nodes that could intersect the target was generated by the system. The optimize for coverage (OCO) objective function was selected, and the required dose was set by the planner. Relaxation values of 20–100 cGy were given to the optimizer. Dose control shells were typically added at 2 mm, 5 mm, and 20 mm for these intracranial treatments. These shells were typically pushed until target coverage was lost to a state of ALARA (as low as can be reasonably achieved). The nearby OARs were added as hard constraints in the optimizer and were pushed to a state of ALARA. A small region of interest was selected for optimization and to assist calculation times. The optimizer was typically performed in high‐resolution mode, where it selected a suitable set of beams from the beamset to achieve the parameters requested in optimization. Multiple optimizations were performed by the planner to ensure acceptable target coverage, and both the OARs and dose control rings were pushed ALARA. The plan was then calculated using the full calculation grid box size and in high resolution. A strict pre‐approval checklist was conducted, and dose‐volume histogram (DVH) plan evaluation and dose reporting sheet were completed. Finally, the treatment plan was signed off by the radiation oncologist.

### 2.3 ICRU 91 reporting metrics

ICRU 91 recommends reporting dose in the PTV, and if applicable in the CTV and/or GTV. As an alternative to the minimum absorbed pixel dose (Min pixel) and Max pixel dose, it is recommended to report the near‐minimum and near‐maximum absorbed doses. These recommendations are made to ensure that the dose reported is not reliant on a single computation point, in which case can suffer from sampling errors and calculation uncertainties. For PTV volumes greater than or equal to 2 cm^3^, $D_{\mathrm{near}-\min}$ is given by $D_{98\%}$, whereas $D_{\mathrm{near}-\max}$ is given by $D_{2\%}$. For PTV volumes less than 2 cm^3^, $D_{\mathrm{near}-\min}$ and $D_{\mathrm{near}-\max}$ are reported by $D_{V-35{\;\mathrm{mm}}^{3}}$ and $D_{35{\;\mathrm{mm}}^{3}}$ dose‐volume metrics, respectively. The reason why the percentage volume elements are not chosen for these smaller PTV volumes is because the percentage specification may lead to volumes that are below the resolution of the dose‐calculation grid. $D_{50\%}$ (i.e., the median dose) is the dose received by at least 50% of the target volume and is likely to be a good measure for the typical absorbed dose in a relatively homogenously irradiated tumor. The rationale behind reporting $D_{50\%}$ is that it is largely representative of the absorbed dose to the PTV.^1^

Radiation‐induced complications associated with ST likely occur because of the dose delivered to the fall‐off region beyond the prescription isodose,^1^ and so the dose gradient is an important quality of a treatment plan to consider. The GI metric intends to provide an objective measure of the dose gradient away from the target volume, and is defined as follows^11^:

(1) $$\mathrm{GI}=\frac{{\mathrm{PIV}}_{\mathrm{half}}}{\mathrm{PIV}}$$

PIV_half_ is the volume receiving at least half the PD, and PIV (prescription isodose volume) is the volume receiving at least the full PD. Lower values of the index indicate steeper gradients.

The dose conformity characterizes the degree to which the high dose region conforms to the target volume. Conformity indices intend to provide an objective measure of a plan's conformity and multiple versions of them have been proposed.^12^, ^13^ ICRU 91 recommends reporting the reciprocal of the Paddick Conformity Index, defined as follows^14^:

(2) $$\mathrm{CI}=\frac{\mathrm{TV}\;\times \;\mathrm{PIV}}{{\mathrm{TV}}_{\mathrm{PIV}}^{2}}$$

where TV is the target volume and TV_PIV_ is the volume of the target covered by at least the PD. This metric considers the location and shape of the prescription isodose. Values closer to unity indicate a more conformal plan.

### 2.4 Treatment plan analysis

#### 2.4.1 Data collection and statistical analysis

**FIGURE 2:**
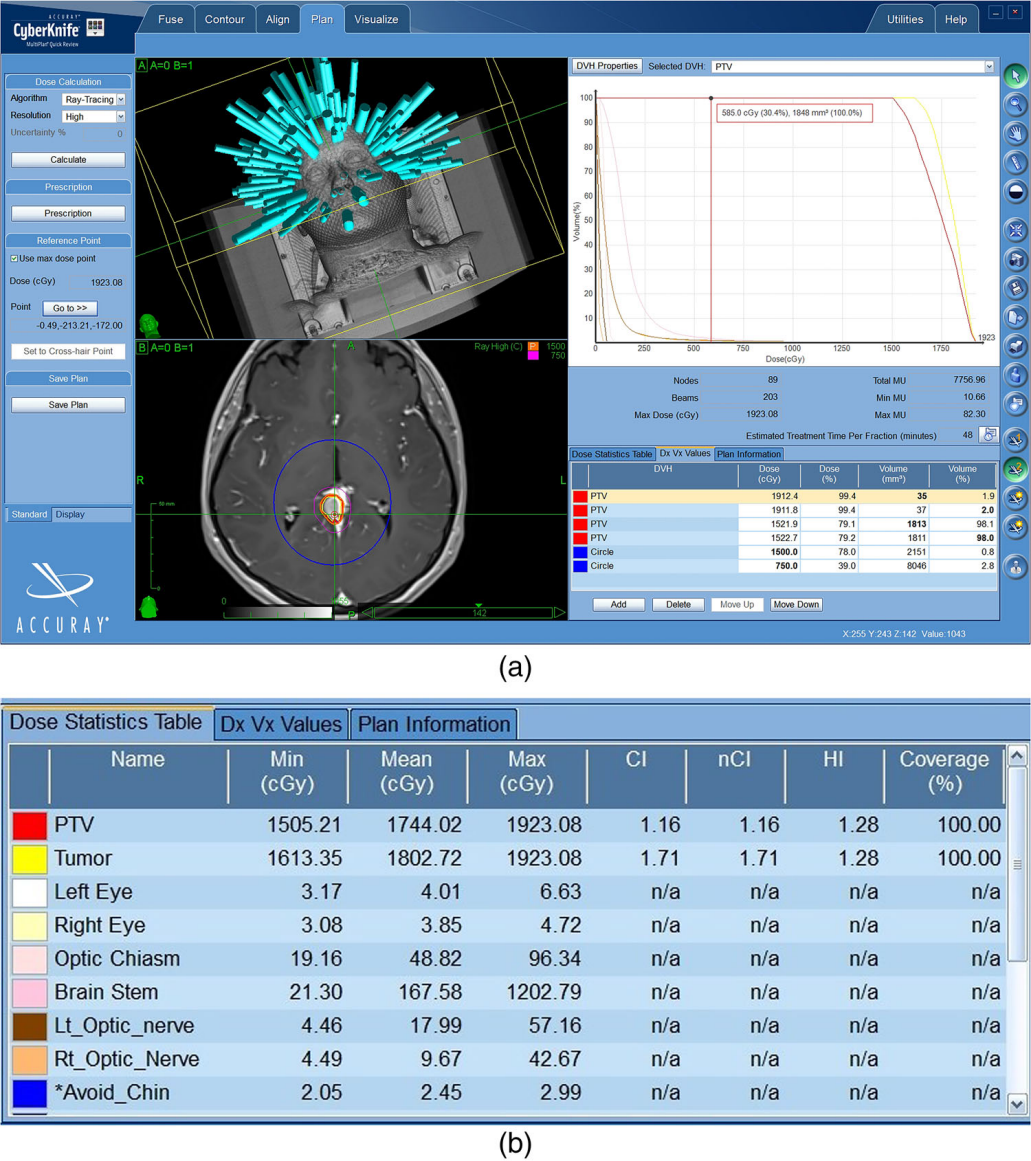
(a) The CyberKnife's treatment planning system (i.e., MultiPlan) with an example meningioma case, with a prescription dose of 1500 cGy, selected. The dose‐volume metrics tab (i.e., Dx Vx Values tab) is selected in the lower right corner. The isodose lines for the prescription dose (orange) and half the prescription dose (i.e., 750 cGy) (pink) are displayed in the MRI transverse view, along with the cylindrical structure (i.e., Circle structure) (blue), contoured into the plan to ultimately determine the GI. (b) Dose Statistics Table from the selected treatment plan.

Each treatment plan was loaded in MultiPlan wherein the values for $D_{\mathrm{near}-\min}$, $D_{\mathrm{near}-\max}$, and $D_{50\%}$ were obtained from the dose‐volume metrics tab (i.e., Dx Vx Values tab) which is displayed in Figure 2a. The CI was obtained from the dose statistics table, shown in Figure 2b. Given that the GI was not provided in the dose statistics table, the metric was obtained by dividing the PIV_half_ and PIV volumes. If these volumes are completely within a structure, then entering the absolute value of the PD and half the PD into that structure's entry in the Dose (cGy) column of the Dx Vx Values tab will provide you with the PIV and PIV_half_ in the Volume (mm^3^) column, respectively. In this study, a cylindrical structure (e.g., the blue Circle structure in Figure 2a) was used to determine the PIV and PIV_half_ volumes. The structure was contoured into each plan, and was ensured to encompass the PIV and PIV_half_ volumes, in order to determine these volumes from the Dx Vx Values tab. Taking the example MEN plan in Figure 2, which had a PD of 1500 cGy, required entering the dose values of 1500 cGy and 750 cGy into the dose column, to obtain the volumes $V_{1500\mathrm{cGy}\;}$(i.e., PIV) and *V*_750cGy_ (i.e., PIV_half_) from the volume column.

As a means of conducting a detailed analysis, the following parameters were also collected: PD, number of fractions, dose per fraction, target volume, maximum target dimension in the x, y, and z direction, Min pixel, Max pixel, homogeneity index (HI) (i.e., the ratio of the Max pixel to the PD), number of treatment beams (NOB), monitor units (MUs), number of nodes, PIDL, and target coverage.

To scale dose values from different PD regimens equivalently, all dose metrics were normalized to the PD of each plan, given as a percentage.

Assessment of the correlations between the ICRU 91 metrics and plan parameters, in each plan group, was conducted using a Pearson correlation analysis, by means of the Pearson correlation coefficient (PCC). The correlations were tested for significance; correlations obtaining *p*‐values less than 0.05 were considered statistically significant. The PCCs and their corresponding *p*‐values were obtained using the pearsonr function from the SciPy stats python library.

#### 2.4.2 Volume‐based analysis for the meningioma and acoustic neuroma treatment plans

As a way of assessing the relationships between the ICRU 91 reporting metrics and other plan parameters within different target volume ranges in the MEN group, a further Pearson correlation analysis was conducted with the treatment plans arranged into small (Group I: <2 cm^3^), medium (Group II: 2–10 cm^3^), and large (Group III: ≥10 cm^3^) volume groups. The small category was selected as per the ICRU 91 small target criteria, the medium category represents the typical MEN tumor volume presented at HMC, and the large category represents tumor volumes typically chosen for fractionated treatments.

A volume‐based Pearson correlation analysis was also performed for the AN group, with plans separated into two groups of target volumes less than and greater than 2 cm^3^.

#### 2.4.3 Combined volume‐based analysis and overall analysis

Following the individual analysis of all three treatment sites, the data were combined to conduct a further volume‐based Pearson correlation analysis, with the 180 treatment plans sorted into three volume groups.

The TGN plans were considered separately (i.e., Group A: target volume <0.1 cm^3^). The remaining 120 plans from the MEN and AN groups were then categorized into the following volume groups: Group B (0.1–2 cm^3^) and Group C (≥2 cm^3^). Group B and C comprised 48 and 72 plans, respectively.

Furthermore, the data from all three treatment sites (*n* = 180) was combined to carry out a Pearson correlation analysis of all collected data.

## 3 RESULTS

### 3.1 $D_{\mathrm{near}-\min}$, $D_{\mathrm{near}-\max}$, and $D_{50\%}$ metrics

#### 3.1.1 Trigeminal neuralgia treatment plans

A summary of the $D_{\mathrm{near}-\min}$, $D_{\mathrm{near}-\max}$, and $D_{50\%}$ metrics in the TGN plans is provided in Table 3. Given that all TGN targets had a volume less than 2 cm^3^, and following the ICRU 91 guidelines, the $D_{\mathrm{near}-\min}$ and $D_{\mathrm{near}-\max}$ were reported according to $D_{V-35{\;\mathrm{mm}}^{3}}$ and $D_{35{\;\mathrm{mm}}^{3}}$, respectively. The $D_{\mathrm{near}-\min}$ was found to be larger than the $D_{\mathrm{near}-\max}$ in 42 out of the 60 TGN plans, whereas in 17 plans both metrics were not applicable.

**TABLE 3:** Summary of the $D_{\mathrm{near}-\min}$,$\;D_{\mathrm{near}-\max}$, and $D_{50\%}\;$ICRU 91 reporting metrics in the trigeminal neuralgia plans

ICRU 91 reporting metric	Range (%)	Mean (median) (%)
$D_{\mathrm{near}-\min}$	113.08–136.04	121.89 (121.21)
$D_{\mathrm{near}-\max}$	100.86–118.36	107.27 (106.14)
$D_{50\%}$	108.23–125.74	115.07 (114.73)

$D_{\mathrm{near}-\min}$ and $D_{\mathrm{near}-\max}$ values were collected from 43 treatment plans, whereas $D_{50\%}$ values were collected from all 60 plans in the group.

*Note*: Doses are normalized to the prescription dose.

When the TGN target volume was between 35–70 mm^3^, $D_{V-35{\;\mathrm{mm}}^{3}}$ resulted in the reporting of *D*_x_, where ‘x’ is an absolute volume less than 35 mm^3^, and so $D_{\mathrm{near}-\min}$ (i.e., *D*_x_) was larger than $D_{\mathrm{near}-\max}$ (i.e., $D_{35{\;\mathrm{mm}}^{3}}$) in these cases. When the target volume was less than 35 mm^3^, $D_{V-35{\;\mathrm{mm}}^{3}}$ gave a dose‐volume metric with a negative volume, and hence $D_{\mathrm{near}-\min}$ did not apply. Similarly, in these plans no $D_{\mathrm{near}-\max}$ was reported since $D_{35{\;\mathrm{mm}}^{3}}$ did not apply. In the single plan with a target volume greater than 70 mm^3^, $D_{\mathrm{near}-\min}$ = $D_{y}$, where ‘*y*’ is an absolute volume greater than 35 mm^3^, and thus the reported $D_{\mathrm{near}-\min}$ was less than $D_{\mathrm{near}-\max}$.

#### 3.1.2 Meningioma treatment plans

A summary of the $D_{\mathrm{near}-\min}$, $D_{\mathrm{near}-\max}$, and $D_{50\%}$ metrics in the MEN analysis is provided in Table 4. Upon further analysis of the sorted MEN volume groups, PCCs were obtained between the three dose metrics and target volume and are presented in Table 4.

**TABLE 4:** Summary of the $D_{\mathrm{near}-\min}$, $D_{\mathrm{near}-\max}$, and $D_{50\%}\;$ICRU 91 reporting metrics in the meningioma treatment plans. Pearson correlation coefficients (and their *p*‐values) between the metrics and target volume are also provided for the entire volume range and in volume groups I, II, and III

ICRU 91 reporting metric	Range (%)	Mean (median) (%)
$D_{\mathrm{near}-\min}$	93.14–111.12	101.26 (101.38)
$D_{\mathrm{near}-\max}$	118.67–130.07	124.72 (123.98)
$D_{50\%}$	107.55–120.91	114.19 (114.09)

		Group I (<2 cm^3^)	Group II (2–10 cm^3^)	Group III (≥10 cm^3^)
**Correlations**	**Target volume**	**Target volume**	**Target volume**	**Target volume**
$D_{\mathrm{near}-\min}$	−0.150 (0.254)	−0.618 (0.011)*	0.059 (0.754)	−0.084 (0.786)
$D_{\mathrm{near}-\max}$	−0.193 (0.140)	−0.221 (0.411)	0.128 (0.494)	0.414 (0.160)
$D_{50\%}$	−0.427 (0.001)*	−0.364 (0.165)	0.117 (0.533)	−0.150 (0.625)

*Note*: Doses are normalized to the prescription dose.

* Statistically significant.

#### 3.1.3 Acoustic neuroma treatment plans

Table 5 presents a summary of the $D_{\mathrm{near}-\min}$, $D_{\mathrm{near}-\max}$, and $D_{50\%}$ metrics from the AN analysis. High and moderate correlations were found between the metrics and target volume in AN targets below 2 cm^3^, while above this volume this was not the case (see Table 5).

**TABLE 5:** Summary of the $D_{\mathrm{near}-\min}$, $D_{\mathrm{near}-\max}$, and $D_{50\%}\;$ICRU 91 reporting metrics in the acoustic neuroma treatment plans. Pearson correlation coefficients (and their *p*‐values) between the metrics and target volume are also provided for the entire volume range and in volumes above and below 2 cm^3^.

ICRU 91 reporting metric	Range (%)	Mean (median) (%)
$D_{\mathrm{near}-\min}$	90.99–108.88	100.11 (100.15)
$D_{\mathrm{near}-\max}$	120.46–141.07	128.69 (126.57)
$D_{50\%}$	109.96–126.91	116.12 (115.17)

Correlations	Target volume	Target volume (<2 cm^3^)	Target volume (≥2 cm^3^)
$D_{\mathrm{near}-\min}$	−0.153 (0.242)	−0.763 (<0.001)*	0.113 (0.566)
$D_{\mathrm{near}-\max}$	−0.491 (<0.001)*	−0.611 (<0.001)*	0.003 (0.988)
$D_{50\%}$	−0.366 (0.004)*	−0.530 (0.002)*	0.128 (0.517)

*Note*: Doses are normalized to the prescription dose.

* Statistically significant.

#### 3.1.4 Combined volume‐based and overall analysis

The PCCs obtained for $D_{\mathrm{near}-\min}$ and $D_{\mathrm{near}-\max}$ with target volume within Group B and C are provided in Table 6. Both dose metrics correlated with target volume only in the smaller tumor plans, where they tended to increase with decreasing volume; in the larger tumor plans the metrics appear to become independent of volume.

**TABLE 6:** Pearson correlation coefficients (and their *p*‐values) between ICRU 91 reporting metrics and target volume and prescription isodose line in treatment plans within volume groups B and C

ICRU 91 reporting metric	Group B (0.1–2 cm^3^) Target volume	Group C (≥2 cm^3^) Target volume
$D_{\mathrm{near}-\min}$	−0.637 (<0.001)*	0.062 (0.603)
$D_{\mathrm{near}-\max}$	−0.522 (<0.001)*	−0.100 (0.405)
GI	−0.503 (<0.001)*	−0.342 (0.003)*
CI	−0.361 (0.013)*	0.051 (0.674)
	PIDL	PIDL
CI	−0.429 (0.003)*	−0.264 (0.026)*

Abbreviation: PIDL, prescription isodose line.

* Statistically significant.

**FIGURE 3:**
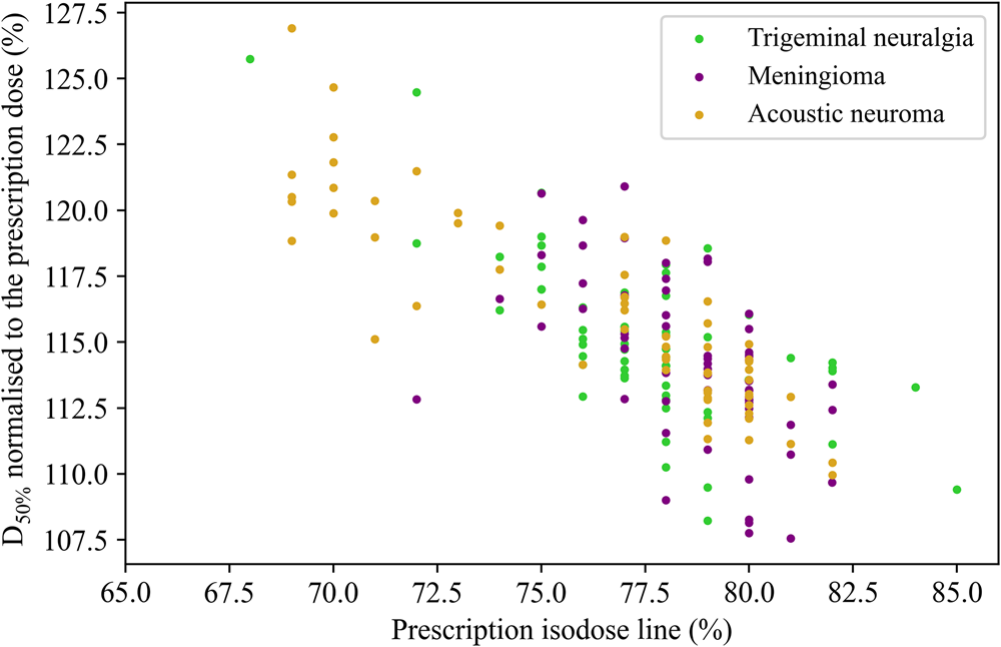
Plot of $D_{50\%}$ versus prescription isodose line from the overall analysis (*n* = 180), where the doses on the y‐axis are normalized to the prescription dose. A Pearson correlation coefficient (PCC) equal to −0.758 (*p*‐value: < 0.001) was obtained between the variables.

Figure 3 presents $D_{50\%}$ as a function of PIDL for all 180 plans analyzed, where a high negative correlation (PCC = −0.758, *p*‐value: < 0.001) was found.

### 3.2 GI metric

#### 3.2.1 Trigeminal neuralgia treatment plans

Table 7 provides a summary of the GI metric from the TGN analysis. A low negative correlation was found between GI and target volume (see Table 7). Furthermore, a moderate positive correlation was found between the GI and PIDL (see Table 7), and thus treatment plans prescribed to lower PIDLs tended to report smaller GI values. The plan prescribed to the lowest PIDL (i.e., 68%) presented the smallest GI (i.e., 4.91) in the plan group.

**TABLE 7:** Summary of the GI and CI metrics in the trigeminal neuralgia treatment plans. Pearson correlation coefficients (and their *p*‐values) between the metrics and plan parameters are also provided.

ICRU 91 reporting metric	Range	Mean (median)
GI	4.91‐8.53	6.90 (6.92)
CI	1.12‐2.38	1.65 (1.65)

Correlations	Target volume	PIDL	NOB	MUs
GI	−0.326 (0.011)	0.573 (<0.001)*	0.254 (0.051)	−0.122 (0.355)
CI	−0.369 (0.004)*	−0.001 (0.992)	−0.385 (0.002)*	−0.202 (0.122)

Abbreviations: NOB, number of treatment beams; PIDL, prescription isodose line.

* Statistically significant.

#### 3.2.2 Meningioma treatment plans

A summary of the GI from the MEN analysis is presented in Table 8. In addition, Table 9 provides the PCCs that the GI obtained with target volume and PIDL within each MEN volume group.

**TABLE 8:** Summary of the GI and CI metrics in the meningioma treatment plans. Pearson correlation coefficients (and their *p*‐values) between the metrics and plan parameters are also provided.

ICRU 91 reporting metric	Range	Mean (median)
GI	2.69–5.14	3.61 (3.45)
CI	1.05–1.76	1.30 (1.26)

Correlations	Target volume	PIDL	NOB	MUs
GI	−0.432 (0.001)*	−0.145 (0.270)	−0.525 (<0.001)*	−0.374 (0.003)*
CI	−0.024 (0.857)	−0.307 (0.018)*	−0.272 (0.037)*	0.285 (0.029)*

Abbreviations: NOB, number of treatment beams; PIDL, prescription isodose line.

* Statistically significant.

**TABLE 9:** Pearson correlation coefficients (and their *p*‐values) between ICRU 91 reporting metrics and target volume and prescription isodose line in each volume group of the meningioma analysis

ICRU 91 reporting metric	Group I (<2 cm^3^)	Group II (2–10 cm^3^)	Group III (≥10 cm^3^)
	Target volume	Target volume	Target volume
GI	−0.405 (0.120)	0.010 (0.958)	−0.054 (0.860)
CI	−0.616 (0.011)*	0.081 (0.672)	0.253 (0.405)
	PIDL	PIDL	PIDL
GI	−0.066 (0.808)	−0.315 (0.084)	0.369 (0.215)
CI	−0.439 (0.089)	−0.290 (0.120)	−0.203 (0.506)

Abbreviation: PIDL, prescription isodose line.

* Statistically significant.

A low negative correlation was found between GI and target volume (see Table 8). The two plans with the largest GI values of 4.97 and 5.14 had target volumes of 1.66 cm^3^ and 0.39 cm^3^, respectively.

Treatment plans which employed a larger NOB tended to report lower GI values (see Table 8), where the plan with the lowest NOB (i.e., 97) presented the highest GI (i.e., 5.14) in the group.

#### 3.2.3 Acoustic neuroma treatment plans

For one plan in the AN analysis, an incorrect entry for the PIV during data collection led to atypical GI and CI values being recorded for this case, and so it was removed from the subsequent GI and CI analysis.

Table 10 provides a summary of the GI metric from the AN analysis. The GI was found to highly correlate with target volume in these plans (see Table 10).

**TABLE 10:** Summary of the GI and CI metrics in the acoustic neuroma treatment plans. Pearson correlation coefficients (and their *p*‐values) between the metrics and plan parameters are also provided.

ICRU 91 reporting metric	Range	Mean (median)
GI	2.52‐4.99	3.80 (3.82)
CI	1.06‐2.18	1.31 (1.30)

Correlations	Target volume	PIDL	NOB	MUs
GI	−0.793 (<0.001)*	−0.457 (<0.001)*	−0.446 (<0.001)*	−0.748 (<0.001)*
CI	−0.451 (<0.001)*	−0.573 (<0.001)*	−0.373 (0.004)*	−0.446 (<0.001)*

Abbreviations: NOB, number of treatment beams; PIDL, prescription isodose line.

* Statistically significant.

The plan which employed the largest NOB, as well as the largest number of MUs, reported the lowest GI in the group.

#### 3.2.4 Combined overall analysis

**FIGURE 4:**
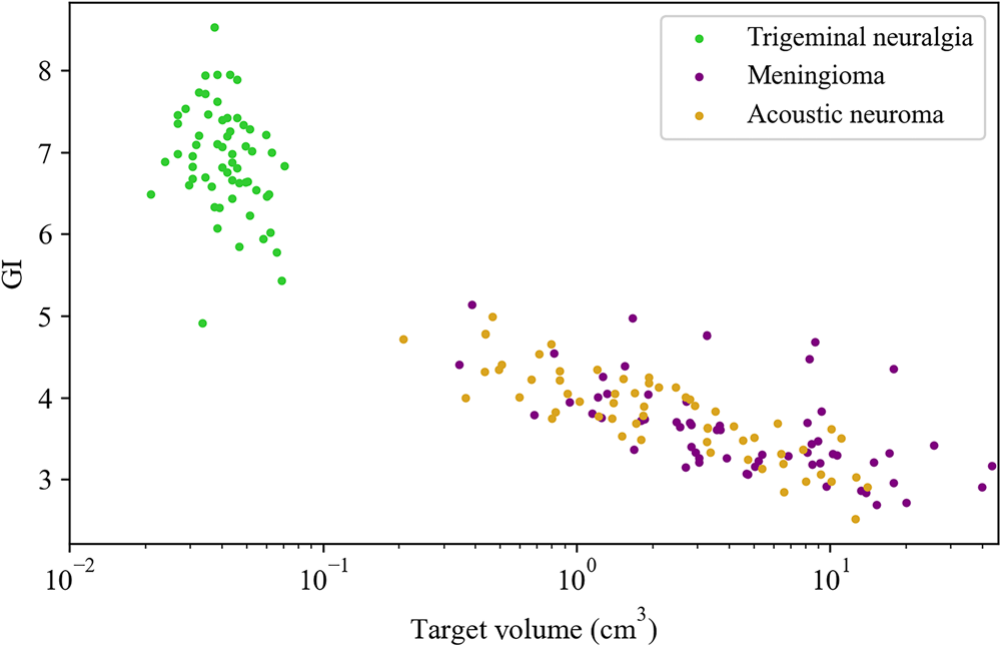
Plot of GI vs target volume from the overall analysis with the x‐axis provided in a logarithmic scale. A Pearson correlation coefficient (PCC) equal to −0.935 (*p*‐value: < 0.001) was obtained between the variables. Note, one outlier was removed from the analysis, where the outlier was a result of incorrect data entry during collection.

The GI as a function of target volume for the entire collection of analyzed plans is presented in Figure 4, where the x‐axis is provided in a logarithmic scale. A PCC of −0.935 (*p*‐value: < 0.001) was obtained between GI and the logarithm of target volume, indicating a very high correlation. Hence, the relationship between GI and target volume is best fitted with the logarithmic function as follows:

(3) $$\mathrm{GI}=-0.679\;\ln\left(\mathrm{TV}\right)+4.551$$

### 3.3 CI metric

#### 3.3.1 Trigeminal neuralgia treatment plans

A summary of the CI metric in the TGN plans is provided in Table 7. The CI yielded a low negative correlation with target volume (see Table 7) in these plans.

#### 3.3.2 Meningioma treatment plans

A treatment plan reporting a CI of 2.42 was considered an outlier, and upon further analysis, it was determined that the target in this case was located adjacent to an air cavity; this ultimately resulted in the plan attaining a relatively large CI. Hence, this plan was removed from the remainder of the CI analysis as it is not considered a typical MEN case presented at HMC and would only skew the findings.

Table 8 provides a summary of the CI from the MEN analysis, whereas the PCCs that the CI obtained with target volume and PIDL within each MEN volume group are provided in Table 9.

A low negative correlation was found between CI and PIDL (see Table 8), with larger CI values generally being reported in plans with lower PIDLs.

#### 3.3.3 Acoustic neuroma treatment plans

A summary of the CI from the AN analysis is provided in Table 10. The CI presented a low negative correlation with target volume (see Table 10). Moreover, the treatment plan with the largest CI (i.e., 2.18) had a target volume of 0.36 cm^3^, which was the second smallest volume in the AN group.

#### 3.3.4 Combined volume‐based analysis

The PCCs between CI and target volume in Group B and C are provided in Table 6. Only in the plans with smaller targets did the CI significantly correlate with target volume, in an inverse manner, while the variables were effectively independent in the larger group (see Table 6). Furthermore, CI significantly correlated with PIDL in both Group B and C, where CI tended to increase in plans with lower PIDLs (see Table 6).

## 4 DISCUSSION

The analysis of the ICRU 91 $D_{\mathrm{near}-\min}$ and $D_{\mathrm{near}-\max}$ metrics in the TGN treatment plans highlighted that these dose metrics break down for the tiny target volumes presented in TGN cases, where $D_{\mathrm{near}-\min}$ was larger than $D_{\mathrm{near}-\max}$ in 42 out of the 60 analyzed plans, and in 17 plans both metrics did not apply. The breakdown of the metrics simply originates from the $D_{V-35{\;\mathrm{mm}}^{3}}$ and $D_{35{\;\mathrm{mm}}^{3}}$ methodology provided in ICRU 91. As the target volume decreases, the percentage of the volume which the dose‐volume metric $D_{V-35{\;\mathrm{mm}}^{3}}$ applies to reduces, leading to a relatively higher dose in the target being reported for $D_{\mathrm{near}-\min}$. Moreover, as the target volume reduces, the percentage of the volume which $D_{35{\;\mathrm{mm}}^{3}}$ applies to increases, and so a relatively lower dose in the target is reported for $D_{\mathrm{near}-\max}$.

Although TGN is commonly treated with SRS, something acknowledged by ICRU 91, the report's recommended near‐minimum and near‐maximum dose metrics are invalid for these treatments. It would be useful if this was addressed in ICRU 91, and if a special category for targets below 0.1 cm^3^ was provided. Though reporting a dose from a single computation point is typically not advised, it is proposed that for TGN treatments the Min and Max pixel are used as alternatives to $D_{\mathrm{near}-\min}$ and $D_{\mathrm{near}-\max}$.

It is evident from the analyses that the use of the $D_{50\%}$ metric for clinical treatment planning is quite limited, though given that it's considered a reliable measure of the typical absorbed dose in the target, it could be employed as a possible dose prescription method, something originally proposed in ICRU 83.^4^ The metric has also been shown to best correlate with the biological equivalent dose,^15^ and so future prescriptions to the metric seem like an attractive option. Furthermore, the metric is predominantly influenced by the PIDL, where a high negative correlation was observed between the variables. Hence, as an alternative to prescribing to the metric, planners could lower the PIDL, and as such increase the $D_{50\%}$ dose, delivering a larger biological dose to the target. Such techniques would need to be supported by large follow‐up studies before being implemented clinically.

In all three sites analyzed, and in the combined analysis, the GI was found to significantly depend on target volume. A very high correlation was found between the GI and the logarithm of target volume, and thus the relationship is best modeled with a logarithmic function. This volume dependence of the GI has been reported by several studies in the literature. Ohtakara et al.^16^ observed a very high negative correlation between the variables in their analysis of Linac‐Based SRS plans for brain metastases; they too found a logarithmic fitting most appropriate for the relationship. Menon et al.^17^ evaluated 60 Linac‐Based SRS/SRT plans for arteriovenous malformations and reported a negative relationship between GI and target volume.

This inverse relationship between GI and target volume can be explained as follows: recall that GI is a volume‐based metric, computed simply by the ratio of PIV_half_ (i.e., PIV_50%_) and PIV (i.e., PIV_100%_). An equivalent dose fall‐off (e.g., 100% to the 50% isodose) in space has a greater impact on smaller compared to larger target volumes, given that the dose falls off in an inverse square manner.^16^

Given the established target volume dependence of the GI, the metric could potentially be used as a plan evaluation tool during the planning of the target sites analyzed in this study. This would provide planners with an objective method of assessing their plans. Future research would need to be conducted to establish the values of the GI in plan evaluation. Moreover, implementing the metric into plan evaluation would lead to an enhancement of treatment plan quality with regard to the dose gradient.

The GI was found to moderately correlate with PIDL in the TGN plans of this study. Hence, it is proposed that planners initially prescribe to the 75% PIDL in TGN planning to achieve a relatively low GI; this should lead to a decrease in the average GI reported, which is particularly advantageous in these treatments given the high doses employed.

Despite other studies presenting a proportional relationship between GI and PIDL,^18^, ^19^, ^20^ other than in the TGN group, such a trend was not observed in this present study. However, this is probably owed to the target volume having the dominant effect on the GI, and it is expected that for an individual target volume, lower PIDLs will always result in a lower GI. This would support the TGN findings as the planning conditions in these cases, including target volume, can be considered constant. Furthermore, for MEN and AN treatment planning, it is also proposed to lower the PIDL to 75% to provide a superior GI on an individual plan basis.

The GI metric yielded a significant negative correlation with NOB in the MEN and AN groups, which is most likely owed to the steeper dose gradients associated with the use of more treatment beams.^19^ Based on these findings, a minimum of 150 treatment beams (up from the current minimum at HMC of 100 beams) is proposed for future cases.

In general, the CI appears to be only volume‐dependent in smaller targets, where it appears to increase with decreasing target volume. Similar findings were observed by Lomax and Scheib^13^ in their retrospective analysis of 551 Gamma Knife intracranial treatment plans. The authors found the analyzed conformity indices to be independent of volume above 1 cm^3^, while below this size they degraded with decreasing target volume.

The inverse CI and target volume relationship in the plans of smaller targets is probably due to the fact that a small change in absolute volume of the PIV will translate to a larger relative change for a smaller than for a larger target.^21^ Taking the example of a spherical target volume of 0.268 cm^3^ which has a constant 1 mm target volume‐PIV margin, a relatively poor CI of 2 would be obtained, though visually the plan would still be considered highly conformal.^13^

Given its volume dependence, the CI could potentially serve as a plan evaluation tool in TGN planning and in the planning for small MEN and AN tumors, which would ultimately lead to more conformal treatments. Future work would need to be carried out to establish the CI values in plan evaluation.

The negative correlation between CI and PIDL in the tumor cases may be owed to the fact that lower PIDLs have a wider penumbra area. Furthermore, it is evident that the CI in plans of smaller tumors is particularly sensitive to a decrease in PIDL, as the correlation between these variables was highest in Group B of the combined analysis. Therefore, it is important to monitor the CI closely when planning these small volumes.

The CI was found to significantly correlate with the NOB in each of the three plan groups. Thus, an increase in the minimum NOB threshold from 100 to 150, as discussed previously, could serve to reduce the CI in these plans.

## 5 CONCLUSION

This work presents an assessment of the ICRU 91 reporting metrics for their use in clinical treatment planning. The $D_{\mathrm{near}-\min}$ and $D_{\mathrm{near}-\max}$ metrics are misleading in treatment plans of very small volumes (e.g., TGN), and it is recommended that the Min and Max pixel are used instead in such cases. The $D_{50\%}$ metric is of limited use for clinical treatment planning, though it may be useful for proposing new prescriptions. The GI was found to significantly depend on target volume. Hence, the planner should always consider the target volume when assessing the dose gradient quality using the GI. The CI is only volume‐dependent in plans of smaller volumes. The GI and CI could potentially serve as complementary plan evaluation tools during the planning of the treatment sites analyzed in this study, which would ultimately enhance plan quality with regard to dose fall‐off and conformity.

## AUTHOR CONTRIBUTION

All authors contributed equally to this project.

## CONFLICT OF INTEREST STATEMENT

The authors declare no conflicts of interest.
